# The relationship between ambient temperature and fasting plasma glucose, temperature-adjusted type 2 diabetes prevalence and control rate: a series of cross-sectional studies in Guangdong Province, China

**DOI:** 10.1186/s12889-021-11563-5

**Published:** 2021-08-11

**Authors:** Jiali Luo, Guanhao He, Yanjun Xu, Zihui Chen, Xiaojun Xu, Jiewen Peng, Shaowei Chen, Jianxiong Hu, Guiyuan Ji, Tao Liu, Weilin Zeng, Xing Li, Jianpeng Xiao, Lingchuan Guo, Qun He, Wenjun Ma

**Affiliations:** 1 Guangdong Provincial Center for Disease Control and Prevention, Guangdong Provincial Institute of Public Health, No. 160, Qunxian Road, Panyu District, Guangzhou, 511430 Guangdong China; 2 Huashan Town Health Center, Huadu District, Guangzhou, 510880 China; 3 School of Public Health, Southern Medical University, Guangzhou, 510515 China; 4 Guangdong Provincial Center for Disease Control and Prevention, Guangzhou, 511430 China; 5 School of Medicine, Jinan University, Guangzhou, 510632 China

**Keywords:** Ambient temperature, Fasting plasma glucose, T2DM, Prevalence, Glycemic control rate

## Abstract

**Background:**

There existed evidence that type 2 diabetes mellitus (T2DM) prevalence and control rate have seasonal variation. Our study aimed to examine the ambient temperature and fasting plasma glucose (FPG) association and estimate temperature-adjusted T2DM prevalence and control rate.

**Methods:**

Four cross-sectional health surveys with 26,350 respondents were conducted in Guangdong Province from 2007 to 2015. Multistage cluster sampling was used to recruit study participants. The data of demographic characteristics, lifestyle factors, diet and use of hypoglycemic medicine, height, weight, FPG and meteorological information were collected. And an inverse distance-weighted method was employed to estimate daily temperature exposures at the individual’ s residential district/county. Base on World Health Organization 2006 criteria, participants were divided into normal fasting glucose (NFG) participants (*n* = 23,877), known T2DM patients (*n* = 916) and newly detected T2DM patients (*n* = 1557). Generalized additive mixed model was employed to evaluate the nonlinear associations between temperature and FPG among different T2DM subgroups. The T2DM prevalence and control rate were estimated based on temperature-FPG association.

**Results:**

The curves of temperature and FPG were downward parabola for total, NFG and known T2DM groups, while it was “U”-shaped for newly detected T2DM patients. When temperature decreased from 30 °C to 4 °C, the FPG significantly increased 0.24 (95%CI: 0.15, 0.33) mmol/L, 0.10 (95%CI: 0.06, 0.14) mmol/L and 1.34 (95%CI: 0.56, 2.12) mmol/L in total, NFG and known T2DM groups, respectively. Compared to 19 °C, newly detected T2DM patients’ FPGs were increased 0.73 (95%CI: 0.13, 1.30) mmol/L at 4 °C and 0.53 (0.00, 1.07) mmol/L at 30 °C. The model-estimated temperature-adjusted T2DM prevalence had a down and up trend, with 9.7% at 5 °C, 8.9% at 20 °C and 9.4% at 30 °C, respectively. At 5, 10, 15, 20, 25 and 30 °C, the model-estimated temperature-adjusted T2DM control rates were 33.2, 35.4, 38.2, 43.6, 49.1 and 55.2%.

**Conclusion:**

Temperature was negatively associated with FPG for NFG and known T2DM subgroups, while their association was U-shape for newly detected T2DM patients. Hence, the temperature-adjusted T2DM prevalence show a dip/peak pattern and T2DM control rate display a rising trend when temperature increase. Our findings suggest temperature should be considered in T2DM clinic management and epidemiological survey.

**Supplementary Information:**

The online version contains supplementary material available at 10.1186/s12889-021-11563-5.

## Background

Type 2 diabetes mellitus (T2DM) is a metabolic disorder characterized by high blood glucose in the context of insulin resistance and relative insulin deficiency. T2DM has been a very serious public health problem worldwide and accounted for about 90% of diabetes cases. It was estimated that diabetes affected more than 425 million people worldwide in 2017, and 114.4 million diabetes cases were in China [1]. Diabetes is a major contributor to cardiovascular diseases and is the eleventh common cause of disability worldwide [1].

Many risk factors such as heredity, individual life style and insufficient activity are associated with T2DM [2–4]. Environmental factors such as ambient temperature, were related to fasting plasma glucose (FPG). There was much evidence on seasonal variation of FPG. For example, there was a mean 0.6 mmol/L difference in FPG between winter and spring in Southern California [5]. *Gikas* et al. found that mean FPG was higher during cold seasons than warm seasons in a study, with the nadir in August (7.60 mmol/L) and the zenith in February (9.12 mmol/L) [6]. In terms of acute effect of ambient temperature, previous studies have observed a negative relationship between temperature and FPG [5, 7]. Nevertheless, *Li* et al. found that the association between temperature and FPG was U-shaped that FPG level was higher in cold or hot temperature [8].

There were also exited evidence that the prevalence of T2DM and glycemic control rate had seasonal variation. A population-based study in *Csongrad*, Hungary revealed the seasonal variation in the prevalence of T2DM with the peak on March and the trough on August [9]. Several studies also revealed seasonal variation of glycemic control rate for T2DM cases that lower control rate emerged in cold winter [6, 10]. These seasonal variations can be attributed to temperature variation to a certain extent. Despite the confirmed associations between ambient temperature and FPG, previous epidemiological surveys conducted in different regions or seasons did not adjust temperature when computing T2DM prevalence and control rate [11]. In addition, different nutritional prescription and hypoglycemic drug therapy made for diabetic cases by considering temperature-FPG association can help achieve better diabetic clinic care and management.

In order to fill the knowledge gap, in the current study, we examined the associations of ambient temperature with FPG in different subgroups based on a series of cross-sectional surveys, and further estimated temperature adjusted prevalence and control rate of T2DM. Our findings are informative for accurately estimating the prevalence of T2DM in large-scale surveys across different climate zones and seasons, and clinic management of cases with T2DM in different seasons.

## Methods

### Study design and population

The Guangdong Chronic Disease and Risk Factors Surveys are a series of provincially representative surveys, which were conducted by Guangdong Provincial Center for Disease Control and Prevention in 2007, 2010 and 2013 and 2015. These surveys aimed to understand the prevalence trend and risk factors of non-communicable diseases such as hypertension, T2DM, and obesity. The questionnaire survey, anthropometric measurements and laboratory analysis followed the same procedure and method. The data from the four surveys were combined to examine the association between ambient temperature and FPG. All participants agreed to participate and signed informed consents form prior to the surveys. The study was approved by the Ethics Committee of Guangdong Provincial Center for Disease Control and Prevention (Ethical review code: 2019025).

Similar sampling protocols were adopted for the surveys of 2007, 2010 and 2013, which has been described previously elsewhere [12]. Briefly, in each of the surveys, 21 districts or counties in Guangdong province were randomly selected by stratified multistage cluster sampling with probability proportional to size. In the second stage, four neighborhoods or townships from each district or county were selected; In the third stage, three communities or villages from each neighborhood or township were chosen; In the fourth stage, 50 to 100 households from each community or village were randomly sampled; Finally, 1 resident aged ≥18 years from each sampled household was selected using the Kish grid method. If there is no resident ≥18 years in the selected household or if the selected resident did not agree to participate in the survey, the household was replaced with another randomly selected household nearby. Details of sampling methods and survey protocols for the nutrition and health survey conducted in 2015 have been described in the previous study [13]. The sample size and survey site of four surveys were show in Table S1 (Additional file 1).

### FPG measurement and T2DM definition

Participants were asked to fast at least 8 h before blood collection. Fasting blood samples were collected by registered nurses. FPG levels were measured on a Hitachi 7600 automatic biochemical analyzer (Hitachi, Ltd., Tokyo, Japan) using reagents obtained from Wako Pure Chemical Industries *Ltd.* at the National CDC of China. According to World Health Organization 2006 criteria [14], known T2DM patients were defined as physician-diagnosed T2DM (confirmed with medical history). Newly detected T2DM patients were defined a new detection of diabetes with an FPG level of 7.0 mmol/L or over among undiagnosed diabetes (without a history of diabetes and hypoglycemic use), and normal fasting glucose (NFG) participants were defined as participants with an FPG level less than 7.0 mmol/L. The T2DM prevalence was defined as the proportion of known T2DM patients and undiagnosed diabetes with an FPG level of 7.0 mmol/L or over. The glycemic control rate was defined as the proportion of known T2DM patients with FPG less than 7.0 mmol/L [14].

### Data collection

#### Questionnaire survey and anthropometric measurements

Participants were interviewed and provided with onsite health examinations. All interviews and examinations were conducted following standard protocols by physicians who had received specific training for the survey and health examination. Questionnaires were used to collect a wide range of information including demographic characteristics, lifestyle and household location, as previous studies described [12, 13]. Demographic characteristics included age, sex, career, education. Physical activity time was defined as leisure time spend in high intensity sports or moderate intensity exercise, such as running, swimming, doing Tai Chi (in hour/day). Sedentary leisure time was defined as time spent in sedentary activities after work, such as watching TV, reading a newspaper and using a computer (in hour/day). Smoking status was measured by whether smoking in the past or present (yes vs no). Drinking status was defined as whether drinking alcohol in the past 12 months (yes vs no). Height and weight were measured following standard protocols. Body mass index (BMI) was calculated as weight divided by the square of height (in kg/m^2^). The information of using hypoglycemic medicine in known T2DM subgroup was also collected. In addition, the information of the weekly food consumption of grains, vegetable, fruit, meat and family history of diabetes was also collected in the surveys of 2010 and 2015.

#### Meteorological data

Daily meteorological data including daily mean, minimum, maximum temperature (°C), relative humidity (%) and sunlight (hour/day) during 2007–2016 of 86 weather monitoring stations were obtained from the Guangdong Meteorological Service. Daily meteorological data was passed through quality control checks. The completeness of each meteorological data was closely to 99.9%. Our survey sites (district or county) were shown in Table S1 in additional file 1. In order to obtain a more accurate measure of exposure, we used an inverse distance weighted (IDW) method to produce a 1 km × 1 km spatial resolution of daily temperatures, relative humidity and sunlight across Guangdong province. The results of 10-fold cross-validation show good prediction accuracy of the interpolation method for daily mean temperature (*R*^2^ = 0.98, RMSE = 0.82 °C), daily minimum temperature (*R*^2^ = 0.98, RMSE = 1.04 °C), daily maximum temperature (*R*^2^ = 0.98, RMSE = 1.05 °C), relative humidity (*R*^2^ = 0.82, RMSE = 5.10%), sunlight (*R*^2^ = 0.87, RMSE = 1.43 h/day) (Fig. S1 in additional file 1). Then, daily meteorological data of each participant were extracted from the corresponding interpolated grid according to their residential districts/counties. We collected the lags 0–6 day (24-h) daily temperatures at the date of survey.

### Statistical analysis

We described distributions of all variables, continuous variables as the means±SD for normally distributed data and median (25th–75th percentile) for skew distributed data. Categorical variables were expressed as numbers and percentages. T-test (for normally distributed continuous data), Kruskal–Wallis test (for skew distributed continuous data) or *χ*^*2*^ test (for categorical variables) were used to compared the difference between NFG, known T2DM and newly detected T2DM subgroups. A Gaussian generalized additive mixed models was used to investigate the relationship between ambient temperature and FPG in different subgroups after adjusting for covariates. According to the previous study [2, 15], covariates included age, sex, BMI, education, career, physical activity, sedentary leisure times, smoking status, drinking status, humidity and use of hypoglycemic medicine. Each district or county was modelled as a random effect. Daily mean temperature was selected as exposure according to the minimum value of Akaike’ s Information criterion (*AIC*) in the model (see Fig. S2 in the Additional file 1). And daily mean temperature, humidity, age and BMI were fitted using a penalized cubic spline function with a degree of freedom (*df*) of 3. The selection of optimal *df* of daily mean temperature was based on graphic smoothness and minimum value of *AIC* (see Fig. S3 in Additional file 1). The regression model was described as the following.

1 $$ {Y}_{im}={\beta}_{0m}+{\beta}_{1m}\ s\left({X}_{temp},k=3\right)+{\beta}_{2m}\ s\left({X}_{humidity},k=3\right)+{\beta}_{3m}\ s\left({X}_{age},k=3\right)+{\beta}_{4m}\ s\left({X}_{BMI},k=3\right)+{\beta}_{5m}\ {X}_{1i}+\cdots +{\beta}_{nm}\ {X}_{ni}+{\upxi}_{\mathrm{j}}+{\epsilon}_{im} $$

Where *m* represents groups (total/ NFP / newly detected T2DM/diagnosed-T2DM participants), *Y*_*im*_ represents participant’s FPG; *β*_*0m*_ is the overall intercept, *β*_*1m*_…*β*_*nm*_ corresponds to coefficients for covariables. *X*_*temp*_, *X*_*humidity*_, *X*_*age*_, *X*_*BMI*_ and *X*_*1i*_…*X*_*ni*_ denotes covariables. *S*() is a penalized cubic spline function, ξ_j_ is the district/county random effect and *ϵ*_*im*_ is the residual error.

In order to check the magnitude of the association between temperature and FPG differed in subgroups (NFG participants, known T2DM patients and newly detected T2DM patients), we added an interaction term of temperature and T2DM status variable on generalized additive mixed model in total population.

After constructing the model, we obtained the curve between daily mean temperature and the difference of FPG compared to the minimum FPG temperature. In order to quantitatively estimate the association between temperature and FPG, we calculated the difference of FPG comparing the minimum/maximum temperature with the minimum FPG temperature. Based on curve, we could compute the difference of FPG (∆*FPG*_*ijm*_) at different ambient temperature. Temperature-adjusted FPG (FPG_2ijm_) (2), prevalence (Rate_1j_) (3) and glycemic control rate (Rate_2j_) (4) of T2DM can be estimated as the follows:

2 $$ {FPG}_{2ijm}={FPG}_{1im}-\Delta  {FPG}_{ijm} $$

3 $$ {Rate}_{1j}={N}_{1j}/{N}_3 $$

4 $$ {Rate}_{2j}={N}_{2j}/{N}_4 $$

Where *m* corresponds to groups (total/NFG/newly detected T2DM/known T2DM participants); *j* represents reference temperature points (5 °C, 10 °C, 15 °C, 20 °C, 22.5 °C, 25 °C and 30 °C). ∆*FPG*_*ijm*_ corresponds to the difference of FPG at temperature on the survey date compared to reference temperature points. *FPG*_*1im*_ is each participant FPG; *N*_*1j*_ represents the sum of the number of known and newly detected T2DM patients and the number of NFG subgroup with *FPG*_*2ijm*_ level of 7 mmol/L or greater; *N*_*3*_ is the number of total population; *N*_*2j*_ represents the number of known T2DM with a *FPG*_*2ijm*_ less than 7 mmol/L; *N*_*4*_ is the number of known T2DM patients.

In sensitivity analysis, we analysis the association between ambient temperature and FPG at different lags (lag1 to lag6). We further added sunshine and precipitation to the model to test the robustness of that association. we also performed sensitivity analysis using the participants whose has weekly food consumption and family history of diabetes information. We reanalyzed the temperature-FPG relationships among subgroup of NFG and newly detected T2DM when we defined T2DM using both FPG and 2-h plasma glucose rather single FPG. We used R software (version 3.5.1, R foundation for Statistical Computing, Vienna, Austria). All statistical tests were two-sided and *P* values of all statistical analyses less than 0.05 was considered statistically significant. The GAM analysis was performed by using package “*mgcv*”.

## Results

### Characteristics of study participants

Total sample size was 26,350, and 90.6% of participants were NFG; 5.9% of subjects were newly detected T2DM patients and 3.5% were known T2DM patients. Overall, the median age of respondents was 50.1 (40.0–60.1) years known and 44.9% of them were males. Compared with NFG participants, known and newly detected T2DM patients were older and had higher levels in BMI, but lower education attainment. Moreover, there were more nonworkers/houseworkers/retirees, non-smokers and non-drinkers in known and newly detected T2DM patients than NFG participants (Table 1). Other statistical description of FPG and daily mean temperature were shown in Table S2 and Fig. S4 in Additional file 1.

**Table 1:** The characteristic of study participants in the study sample

Variable	Total	Normal fasting glucose^†^	Known T2DM^‡^	newly detected T2DM^§^	*P* value
**Overall, n (%)**	26,350(100.0)	23,877 (90.6)	916 (3.5)	1557 (5.9)	
**Sex, n (%)**					0.2
male	11,834 (44.9)	10,709 (44.9)	398 (43.4)	727 (46.7)	
female	14,516 (55.1)	13,168 (55.1)	518 (56.6)	830 (53.3)	
**Education, n (%)**					**< 0.001**
≤ Junior high school	12,573 (47.7)	11,212 (47.0)	506 (55.2)	855 (54.9)	
High school/college	13,777 (52.3)	12,665 (53.0)	410 (44.8)	702 (45.1)	
**Career, n (%)**					**< 0.001**
production staff	9170 (34.8)	8428 (35.3)	242 (26.4)	500 (32.1)	
Technical staff	9504 (36.1)	8783 (36.8)	224 (24.5)	497 (31.9)	
Nonworkers/houseworkers/retirees	7676 (29.1)	6666 (27.9)	450 (49.1)	560 (36.0)	
**Smoking status, n (%)**					**< 0.001**
Yes (Former/Current)	10,747 (40.8)	9835 (41.2)	357 (39.0)	555 (35.6)	
Never	15,603 (59.2)	14,042 (58.8)	559 (61.0)	1002 (64.4)	
**Drinking status, n (%)**					**< 0.001**
Yes (past 12 months)	11,380 (43.2)	10,412 (43.6)	355 (38.8)	613 (39.4)	
Never (past 12 months)	14,970 (56.8)	13,465 (56.4)	561 (61.2)	944 (60.6)	
**BMI (kg/m**^**2**^**), n (%)**					**< 0.001**
< 18.5	1945 (7.4)	1835 (7.7)	30 (3.3)	80 (5.1)	
18.5–23.9	15,061 (57.2)	13,945 (58.4)	396 (43.2)	720 (46.2)	
24.0–27.9	7280 (27.6)	6384 (26.7)	359 (39.2)	537 (34.5)	
≥ 28	2064 (7.8)	1713 (7.2)	131 (14.3)	220 (14.1)	
**Use of hypoglycemic medicine, n (%)**					–
Yes	676 (2.6)	0	676 (73.8)	0	
Not	25,674 (97.4)	23,877 (100)	240 (26.2)	1557 (100)	
**Age (year), median (25th–75th percentile)**	50.1 (40.0–60.1)	49.8 (39.0–59.9)	59.0 (52.0–67.0)	54.0 (44.8–63.0)	**< 0.001**
**Body mass index (kg/m**^**2**^**), median (25th–75th percentile)**	22.7 (20.6–25.1)	22.6 (20.5–24.9)	24.3 (22.4–26.5)	23.9 (21.5–26.4)	**< 0.001**
Physical activity time (hour/day)*, mean ± SD	0.2 ± 0.7	0.2 ± 0.7	0.3 ± 0.6	0.2 ± 0.5	**< 0.001**
**Sedentary leisure time (hour/day), median (25th–75th percentile)**	4.0 (2.0–6.0)	4.0 (2.0–6.0)	4.5 (3.0–6.8)	4.0 (2.0–6.0)	**< 0.001**
**Daily mean humidity**^**#**^**, median (25th–75th percentile)**	75.0 (66.0–84.0)	75.0 (66.0–84.0)	78.0 (69.0–90.0)	75.0 (64.0–85.0)	**< 0.001**

Data were expressed as median (25th–75th percentile) for non-normal continuous variables and as number (percentage) for categorical variables. Statistics analysis: Kruskal-Wallis test for non-normal continuous variables and chi square test for categorical variables

†: Normal fasting glucose, no medical history of type 2 diabetes mellitus with fasting plasma glucose less than 7.0 mmol/L.

‡: Known T2DM, physician-diagnosed type 2 diabetes mellitus

§: Newly detected T2DM, newly detected type 2 diabetes mellitus with fasting plasma glucose of 7.0 mmol/L or greater

*: Physical activity time variable of median (25th–75th percentile) was zero, so it was expressed as mean ± SD

#: Daily mean humidity was matched by the same date of health survey

### Monthly variation of FPG and ambient temperature

**Fig. 1:**
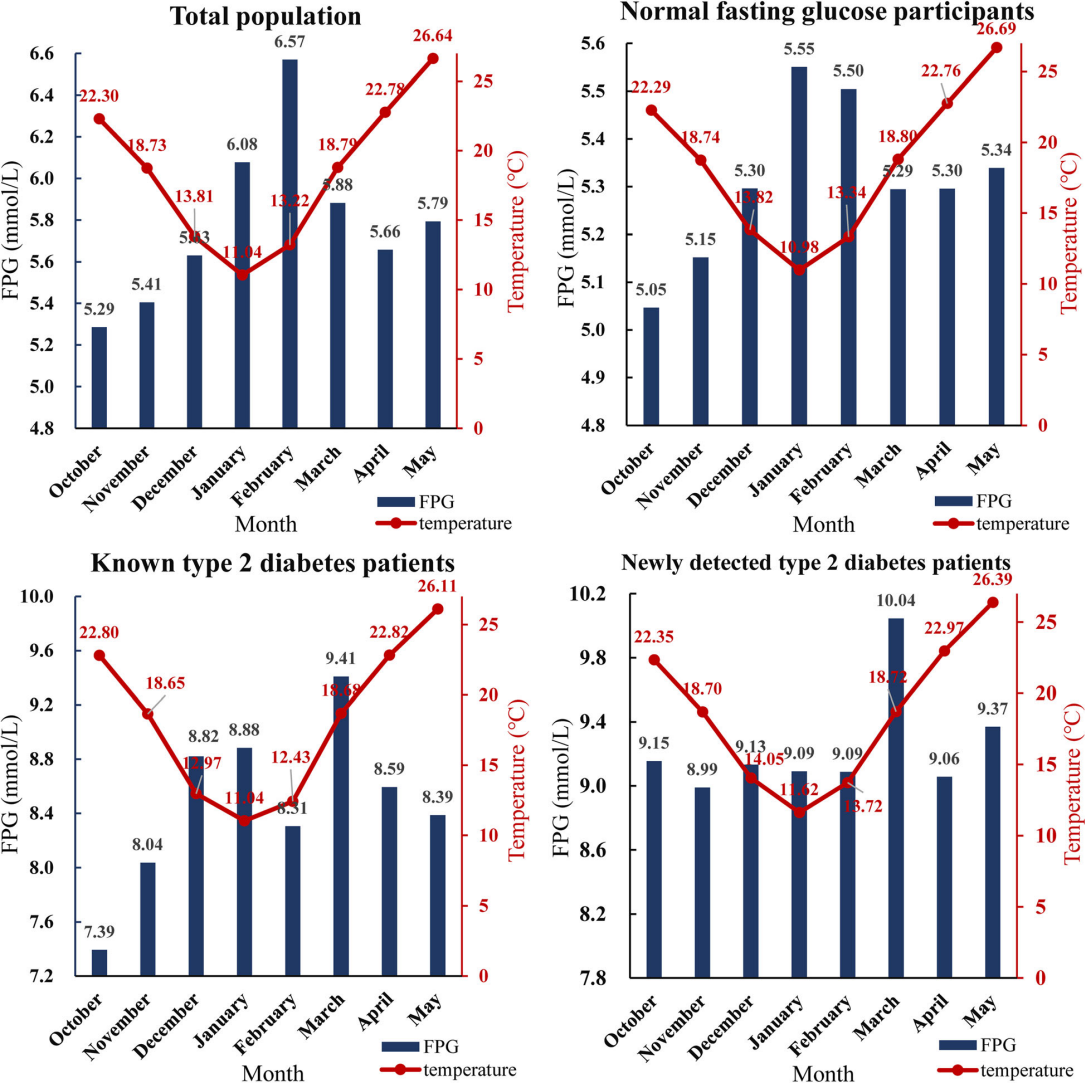
Monthly variation of mean FPG (mmol/L) and ambient temperature (°C) among total population, normal fasting glucose, known T2DM and newly detected T2DM subgroups

Figure 1 and Table S3 (in Additional file 1) show monthly variation of FPG and ambient temperature. The weather factor is characterized by two main seasons: cold season (lasting from December to March) and warm season (April, May, October, November). The mean FPG levels were significantly higher during cold season than warm season. The zenith of FPG was 6.57 mmol/L for total population, 5.55 mmol/L for NFG participants, 9.41 mmol/L for known T2DM patients, and 10.04 mmol/L for newly detected T2DM patients in cold season, while the nadir of FPG was 5.29 mmol/L for total population, 5.05 mmol/L for NFG participants, 7.39 mmol/L for known T2DM patients and 8.99 mmol/L for newly detected T2DM patients in warm season.

### The relationships between ambient temperature and FPG

**Fig. 2:**
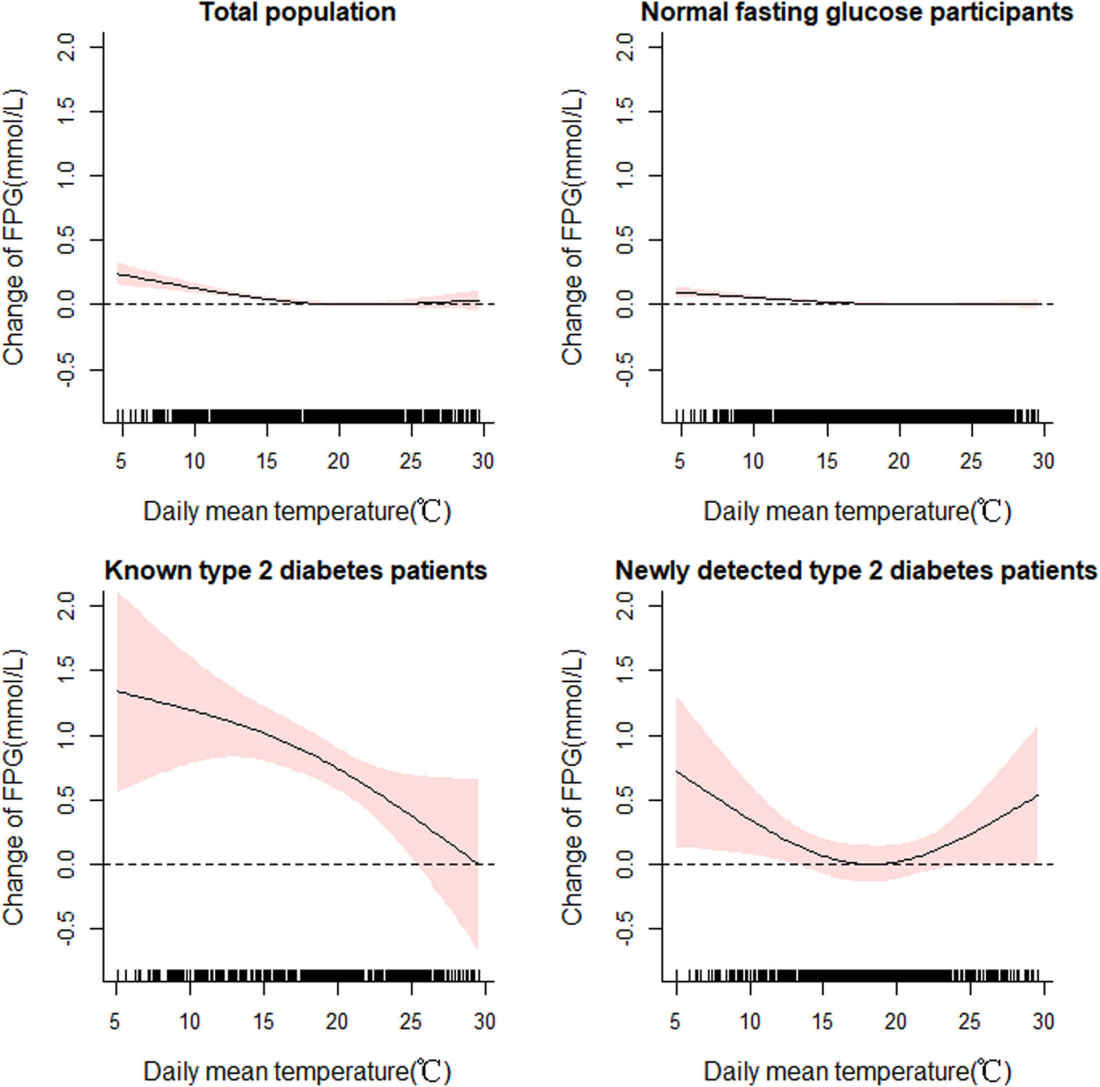
The curves of daily mean temperature and FPG among total population, normal fasting glucose, known T2DM and newly detected T2DM subgroups. Shade represents 95%CI Confidence interval. Gaussian generalized additive mixed models were adjusted for age, sex, BMI, education, career, physical activity, sedentary leisure times, smoking status, drinking status, humidity and use of hypoglycemic medicine variables

In total, NFG and known T2DM groups, the curves were general downward parabola. As temperature increased, FPG concentration significantly decreased. However, the curve was “U” shaped in newly detected T2DM patients (Fig. 2).

When temperature decreased from 30 °C to 5 °C on the survey day (lag 0), the FPG significantly increased 0.24 (95%CI: 0.15, 0.33) mmol/L, 0.10 (95%CI: 0.06, 0.14) mmol/L and 1.34 (95%CI: 0.56, 2.12) mmol/L in total, NFG and known T2DM groups, respectively. For newly detected T2DM patients, when temperature decreased from 19 °C to 4 °C on the survey day (lag 0 day) the FPG significantly increased 0.73 (95%CI: 0.13, 1.30) mmol/L; when temperature increased from 19 °C to 30 °C on lag 0 day, the FPG increased 0.53 (0.00, 1.07) mmol/L (Table 2). Interaction analyses showed that known and newly detected T2DM subgroups’ FPG were more susceptible to ambient temperature than NFG group in Table S4 in Additional file 1 (*P* < 0.001). The temperature and FPG associations were robust to the used for different lag 1–6 days temperature (Table 2).

**Table 2:** Difference of FPG (mmol/L) at different typical interval of temperature

	Total population	Normal fasting glucose participants	Known type 2 diabetes patients	Newly detected type 2 diabetes patients	
Temperature ^a^	5 °C vs 30 °C	5 °C vs 30 °C	5 °C vs 30 °C	5 °C vs 19 °C	30 °C vs 19 °C
Main model					
Lag 0 day	0.24 (0.15, 0.33)	0.10 (0.06, 0.14)	1.34 (0.56, 2.12)	0.71 (0.13, 1.30)	0.53 (0.00, 1.07)
Lag 1 day	0.22 (0.14, 0.29)	0.11 (0.08, 0.15)	1.21 (0.45, 1.97)	0.71 (0.18, 1.24)	0.50 (−0.09, 1.08)
Lag 2 day	0.26 (0.19, 0.34)	0.16 (0.12, 0.19)	0.99 (0.38, 1.61)	0.89 (0.35, 1.42)	0.58 (0.00, 1.15)
Lag 3 day	0.39 (0.32, 0.45)	0.26 (0.23, 0.29)	1.29 (0.56, 2.03)	0.91 (0.23, 1.59)	0.40(−0.14, 0.94)
Lag 4 day	0.37 (0.31, 0.43)	0.23 (0.20, 0.26)	1.50 (0.80, 2.20)	1.02 (0.40, 1.63)	0.55 (0.03, 1.08)
Lag 5 day	0.39 (0.30, 0.47)	0.23 (0.20, 0.27)	1.84 (1.08, 2.60)	0.89 (0.36, 1.42)	0.53 (0.01, 1.05)
Lag 6 day	0.36 (0.31, 0.42)	0.23 (0.19, 0.26)	1.79 (0.96, 2.62)	0.22 (−0.26, 0.70)	0.14 (− 0.30, 0.57)
Model 2	0.25 (0.17, 0.34)	0.14 (0.11, 0.17)	1.20 (0.47, 1.94)	0.70 (0.11, 1.29)	0.49 (0.06, 1.06)

^a^ Represents the difference of FPG when minimum of daily mean temperature (5 °C) compared with threshold temperature (30 °C) in total population, normal fasting glucose and known T2DM subgroups, the difference of FPG when minimum/maximum of daily mean temperature (5 °C/30 °C) compared with threshold temperature (19 °C) in Newly detected type 2 diabetes patients

Main model was adjusted for age, sex, BMI, education, career, physical activity, sedentary leisure times, smoking status, drinking status, humidity and use of hypoglycemic medicine variables. Model2 was adjusted model 1 and additionally sunshine and precipitation variables

### Temperature-adjusted FPG, prevalence and glycemic control rate of T2DM

**Fig. 3:**
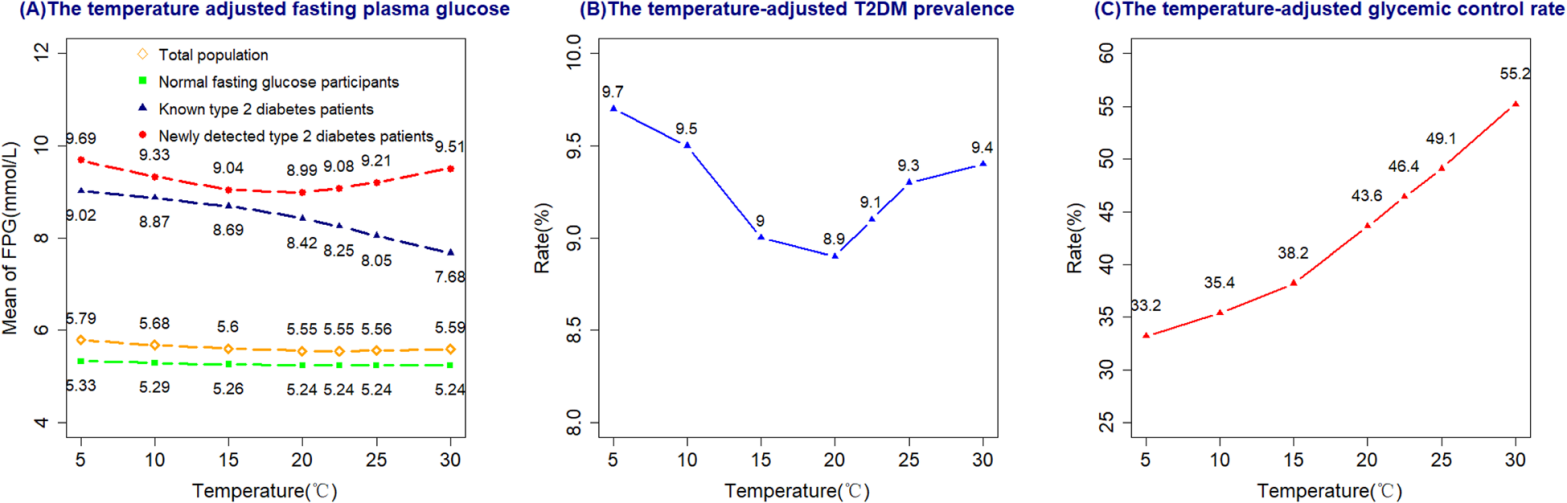
**A** Temperature adjusted FPG concentrations based on temperature-FPG association. **B** The temperature-adjusted T2DM prevalence (%) based on temperature-FPG association. **C** The temperature-adjusted T2DM glycemic control rate (%) based on temperature-FPG association. Adjusted FPG, prevalence and glycemic control rate of T2DM at 5 °C, 10 °C, 15 °C, 20 °C, 22.5^*^°C, 25 °C, 30 °C. (22.5 °C is the annual mean temperature in Guangdong Province)

With the increase of temperature, the FPG of total and NFG groups were changed a little bit. However, temperature adjusted FPG greatly decreased when temperature increased for known T2DM group. For instance, the mean of temperature-adjusted FPG was 9.02 mmol/L at 5 °C while 7.68 mmol/L at 30 °C in known T2DM patients. The association between temperature and FPG was a down and up trend in newly detected T2DM patients. For instance, the mean of temperature-adjusted FPG was 9.69 mmol/L at 5 °C, 8.99 mmol/L at 20 °C and 9.51 mmol/L at 30 °C, respectively (Fig. 3A).

Based on temperature-FPG association, we adjusted prevalence and glycemic control rate of T2DM (Fig. 3B,C). The association between temperature and temperature-adjusted T2DM was a down and up trend, with 9.7% at 5 °C, 8.9% at 20 °C and 9.4 at 30 °C, respectively. Low ambient temperature led to greatly decrease in glycemic control rate of T2DM. For example, when ambient temperature decreased from 30 °C to 5 °C, the glycemic control rate of T2DM decreased from 55.2 to 33.2%.

### Sensitivity analyses

Similar curves between temperature and FPG were observed when different lag times were used or additional covariates like sunshine, precipitation, weekly food consumption and family history of diabetes were controlled (Fig. S5, Fig. S6 and Fig. S7 in Additional file 1). Moreover, there were no significant difference of temperature-FPG association compared to the result of the main model analysis, suggesting that our results were relatively robust (Table 2). In addition, similar relationships between temperature and FPG were observed when using FPG and 2-h plasma glucose as diagnosed criteria (Fig. S8 in Additional file 1).

## Discussion

In our study, we found that FPG levels were significantly higher in cold season than warm season in total and different T2DM groups. The curves between ambient temperature and FPG were downward parabola-shaped in total, NFG and known T2DM groups, while it was U-shaped in newly detected T2DM patients. The prevalence and glycemic control rate of T2DM varied across temperature. The association between temperature and temperature-adjusted T2DM was a down and up trend as temperature increased, while glycemic control rate of T2DM increased with the rising of temperature. This is the first large-scale study estimating temperature adjusted prevalence and control rate of T2DM.

We found that FPG had apparent seasonal variation with high concentrations in cold season and low concentrations in warm season, which was consistent with previous studies [5, 6, 16]. For instance, a similar association was observed that the level of FPG was 0.6 mmol/L higher in winter (13 °C) than in summer (23 °C) in San Diego County, California [5]. A large study included 15 middle and high countries also found that higher FPG among adults was observed in winter than summer in both Northern Hemisphere and Southern Hemisphere [16].

Previous studies have reported that a negative association between ambient temperature and FPG [5, 7]. However, the researches just estimated their linear association rather than the non-linear relationship between ambient temperature and FPG, and they did not consider the variation of temperature-FPG associations among various T2DM groups. For total population, the curve of temperature and FPG like downward parabola-shaped, while the Kailuan cohort study found that curves like U-shaped, which may be due to the difference in study areas and populations [8]. For known T2DM patients, FPG variation associated with temperature difference is similar with *Aristofanis*’ study, which found that known T2DM patients’ fasting glucose levels were significantly higher by 1.52 mmol/L in February (5–10 °C) than in August (29–35 °C) [6]. For newly detected T2DM patients, we found that curve was U-shaped. Our finding suggests people with borderline high FPG in moderate temperature could be diagnosed as T2DM in cold temperature. The temperature and FPG curves were different between known T2DM patients and new T2DM patients. This result may be explained by that newly detected T2DM patients were under large fluctuation in FPG without taking hypoglycemic medicine, which may affect the stability of FPG. Future studies were needed to investigate this mechanism of this phenomenon. Secondly, as the present study was conducted in winter, spring and autumn and without high temperature, which could not comprehensively estimate the association between hot temperature and FPG. Future studies conducted in summer can further observe the association of extremely high temperature with FPG. In addition, our results indicated that the influence of temperature on the FPG seems to be stronger in T2DM patients than NFG participants. The reason may be T2DM is associated with declining insulin sensitivity and beta-cell function, and the glucose regulation of T2DM was poorer than NFG participants [17].

Related mechanism on the inverse association between ambient temperature and FPG is that temperature variation may affect glucose homeostasis including plasma insulin and glucagon [18, 19]. Sustained exposure to low ambient temperature increase energy expenditure and insulin resistance [20, 21]. Population study demonstrated that per 10 °C increase of outdoor temperature was associated with 0.57 units increase of insulin sensitivity index [22]. Extreme cold temperature Exposure could lead to increase in the thyrotropin and decrease in total thyroxine and free thyroxine, which related to decreased insulin sensitivity [23, 24].

We further found the association between temperature and temperature-adjusted T2DM prevalence was a down and up trend as temperature increased. To our knowledge, previous T2DM prevalence surveys rarely considered the effects of ambient temperature [11]. Our finding indicates that ignoring the temperature and FPG association in a large-scale survey across different seasons and climatic zones may bias T2DM prevalence.

We further found that T2DM control rate was positively associated with temperature. *Sakamoto* et al. found that T2DM patients’ control rates of glucose was 4.2% higher in summer than winter [10]. Our results confirmed above study, but the control rate changed more dramatically, rising from 33.2 to 55.2% when temperature rose from 5 to 30 °C. Our finding indicates the effective dose of hypoglycemic drugs for T2DM patients at moderate temperature may be insufficient at low temperature, which leads to inadequate control of blood glucose. This finding suggested for diabetologists and dietitians to modify the treatment protocols, nutritional prescription and exercise plans for those diabetic patients during the cold temperature months.

Our study had several strengths. First, our study sample was relatively large. Secondly, we analyzed the associations between FPG and temperature among different T2DM subgroups. Thirdly, we estimated the temperature adjusted T2DM prevalence and control rate. However, several limitations should be warranted. Firstly, since there were many missing data on participants addresses, we used the meteorological data of participants’ residential districts/communities as their temperature exposure, which may not be precise enough. Considering the altitudes of the participant’s residential districts/counties are ranging from 17 m to 403 m, we did not adjust the altitude when we used IDW method to estimated temperature. The results of 10-fold cross-validation show good prediction accuracy of the interpolation method even without altitude. The IDW method has been well applied to interpolate meteorological factors in previous studies [25, 26]. Secondly, the cross-sectional design of the study may restrict our ability to estimate the causal effect of temperature on FPG. However, we respectively used the temperature 0–6 days prior to the measurement of FPG as exposure and got consistent results, which partially guarantee the time sequential relationship between temperature exposure and FPG. Thirdly, since we did not obtain the dietary and hereditary information of all participants, which limited us to control these covariates in the analysis. Instead, we performed sensitivity analysis using the participants who has weekly food consumption of grains, vegetables, fruit, meat and family history of diabetes information. The estimation on the association of temperature and FPG remained relatively stable after controlling for weekly food consumption and family history of diabetes (see Fig. S6 in Additional file 1). Fourth, we only collected data from October to May, which limited us to estimate the associations between temperature and FPG in hot seasons. Further study should be conducted in the future. Fifth, the model fitting did not consider the impact of income status and air pollutants due to too missing values for income or lacking of relevant data for air pollution. Finally, known T2DM patients might drive changes in individual behaviors that might impact exposure to outdoor temperature, so the estimation of temperature and FPG is affected.

## Conclusion

In conclusion, temperature was negatively associated with FPG for NFG and known T2DM subgroups, while their association was U-shape for newly detected T2DM patients. Hence, the temperature-adjusted T2DM prevalence show a dip/peak pattern and temperature-adjusted T2DM control rate display a rising trend when temperature increase. Our findings suggest that temperature should be considered in large-scale epidemiological survey. For clinical implications of T2DM, the temperature-FPG association can help improve the T2DM diagnosis and guide decisions regarding hypoglycemic treatment to achieve better FPG control.

## Supplementary Information

**Additional file 1: Table S1.** Number (%) of participants in each survey location in Guangdong province during 2007 and 2016. **Fig. S1.** Scatter plot of 10-fold cross-validation of interpolated daily temperatures, relative humidity and sunlight. **Fig. S2.** The non-linear relationships between daily mean, daily minimum, daily maximum temperature and FPG in total population and different T2DM subgroups. **Fig. S3.** The non-linear relationships between daily mean and FPG in total population and different T2DM subgroups at k = 3,4,5. **Fig. S4.** The density distribution of FPG in total population and different T2DM subgroups. **Table S2.** Statistical description of FPG and daily mean ambient temperature in total population and different T2DM subgroups. **Table S3.** Number (%), Mean and SE of FPG and daily mean temperature according to month. **Fig. S5**. The relationships between lag1 to lag6 of ambient temperature and FPG in total population and different T2DM subgroups. Table S4. The interaction analyses about the association between temperature and FPG based on effect modifier (T2DM status). **Fig. S6.** The relationships between ambient temperature and FPG in total population and different T2DM subgroups additionally adjusted sunshine and precipitation. **Fig. S7.** The relationships between ambient temperature and FPG in total population and different T2DM subgroups in survey of 2010 and 2015. **Fig. S8.** The relationships between ambient temperature and FPG in normal fasting glucose and newly detected-T2DM subgroups for the diagnostic standard of T2DM based on FPG and 2-h plasma glucose in 2013 survey.

## Data Availability

Data are not publicly available and information on how to access the data can contact details: Email: mawj@gdiph.org.cn.

## Abbreviations

T2DM: Type 2 Diabetes Mellitus
FPG: Fasting Plasma Glucose
NFG: Normal Fasting Glucose
BMI: Body Mass Index
IDW: Inverse Distance Weighted
AIC: Akaike’ s Information Criterion
Df: Degree of Freedom
