# Supplementary material for: The relationship between ambient temperature and fasting plasma glucose, temperature-adjusted type 2 diabetes prevalence and control rate: a series of cross-sectional studies in Guangdong Province, China
Source: BMC Public Health. 2021 Aug 11;21:1534. doi: 10.1186/s12889-021-11563-5 (PMC8356456; doi:10.1186/s12889-021-11563-5)
Supplement: Supplementary file 1 — Additional file 1: Table S1. Number (%) of participants in each survey location in Guangdong province during 2007 and 2016. Fig. S1. Scatter plot of 10-fold cross-validation of interpolated daily temperatures, relative humidity and sunlight. Fig. S2. The non-linear relationships between daily mean, daily minimum, daily maximum temperature and FPG in total population and different T2DM subgroups. Fig. S3. The non-linear relationships between daily mean and FPG in total population and different T2DM subgroups at k = 3,4,5. Fig. S4. The density distribution of FPG in total population and different T2DM subgroups. Table S2. Statistical description of FPG and daily mean ambient temperature in total population and different T2DM subgroups. Table S3. Number (%), Mean and SE of FPG and daily mean temperature according to month. Fig. S5. The relationships between lag1 to lag6 of ambient temperature and FPG in total population and different T2DM subgroups. Table S4. The interaction analyses about the association between temperature and FPG based on effect modifier (T2DM status). Fig. S6. The relationships between ambient temperature and FPG in total population and different T2DM subgroups additionally adjusted sunshine and precipitation. Fig. S7. The relationships between ambient temperature and FPG in total population and different T2DM subgroups in survey of 2010 and 2015. Fig. S8. The relationships between ambient temperature and FPG in normal fasting glucose and newly detected-T2DM subgroups for the diagnostic standard of T2DM based on FPG and 2-h plasma glucose in 2013 survey. [file 12889_2021_11563_MOESM1_ESM.docx]

**Supplemental material**

Table S1. Number (%) of participants in each survey location in Guangdong province during 2007 and 2016.

| Area | y2007 and y2008 | y2010 and y2011 | y2013 and y2014 | y2015 and y2016 |
| --- | --- | --- | --- | --- |
| Total, N (%) | 6354 (100) | 3566 (100) | 8016 (100) | 8414 (100) |
| Huizhou (Boluo), N (%) | 316 (5.0) | — | — | — |
| Chaozhou (Chaoan), N (%) | 281 (4.4) | — | — | — |
| Dongguang (Dongguang), N (%) | 308 (4.8) | — | — | — |
| Zhuhai (Doumen), N (%) | 315 (4.9) | — | — | — |
| Guangzhou (Yuexiu), N (%) | 310 (4.9) | 595 (16.7) | 548 (6.8) | 511 (6.5) |
| Maoming (Huazhou), N (%) | 313 (4.9) | — | — | — |
| Heyuan (Lianping), N (%) | 312 (4.9) | — | — | — |
| Foshan (Nanhai), N (%) | 319 (5.0) | — | — | — |
| Shaogaun (Nanxiong), N (%) | 316 (5.0) | 600 (16.8) | 562 (7.0) | 622 (7.3) |
| Jieyang (Puning), N (%) | 313 (4.9) | — | — | — |
| Shantou (Jingping), N (%) | 203 (3.2) | — | — | — |
| Shanwei (chengqu), N (%) | 309 (4.9) | 579 (16.2) | 531 (6.6) | 614 (7.3) |
| Shenzhen (Nanshan), N (%) | — | — | — | 650 (7.7) |
| Shenzhen (Futian), N (%) | 309 (4.9) | — | — | — |
| Zhaoqing (Sihui), N (%) | 313 (4.9) | 599 (16.8) | 597 (7.4) | 600 (7.1) |
| Jiangmen (Taishan), N (%) | 287 (4.5) | — | — | — |
| Meizhou (Wuhua), N (%) | 313 (4.9) | 593 (16.6) | 593 (7.4) | 548 (6.5) |
| Zhanjiang (Xunwen), N (%) | 306 (4.8) | — | — | — |
| Yangjiang (Yangxi), N (%) | 279 (4.4) | — | — | — |
| Qiangyuan (Yingde), N (%) | 317 (5.0) | — | — | — |
| Yunfu (Yuncheng), N (%) | 300 (4.7) | 600 (16.8) | 566 (7.1) | 586 (7.0) |
| Zhongshan (Zhongshan), N (%) | 315 (5.0) | — | — | — |
| Maoming (Gaozhou), N (%) | — | — | 572 (7.1) | 618 (7.3) |
| Jieyang (Huilai), N (%) | — | — | 591 (7.4) | 620 (7.4) |
| Huizhou (Huiyang), N (%) | — | — | 587 (7.3) | 582 (6.9) |
| Yunfu (Luoding), N (%) | — | — | 529 (6.6) |  |
| Qingyuan (Qingcheng), N (%) | — | — | 589 (7.3) | 621 (7.4) |
| Shaoguan (Qujiang), N (%) | — | — | 586 (7.3) | 610 (7.2) |
| Foshan (Shunde), N (%) | — | — | 585 (7.3) | 621 (7.4) |
| Zhanjiang (Wuchuan), N (%) | — | — | 580 (7.2) | 611 (7.3) |

—, the location was not surveyed in corresponding year and its data was not available


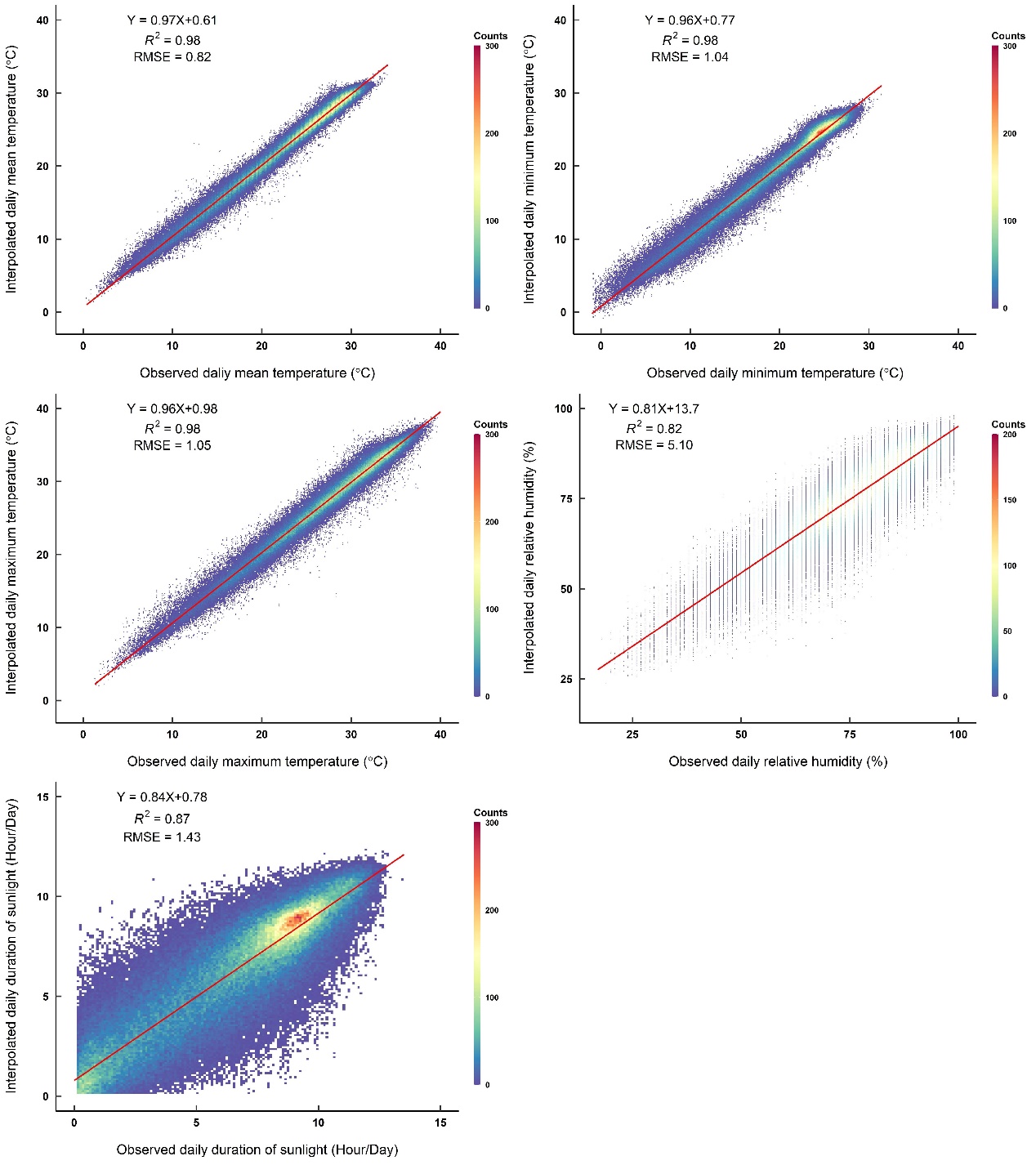


Figure S1 Scatter plot of 10-fold cross-validation of interpolated daily temperatures, relative humidity and sunlight.


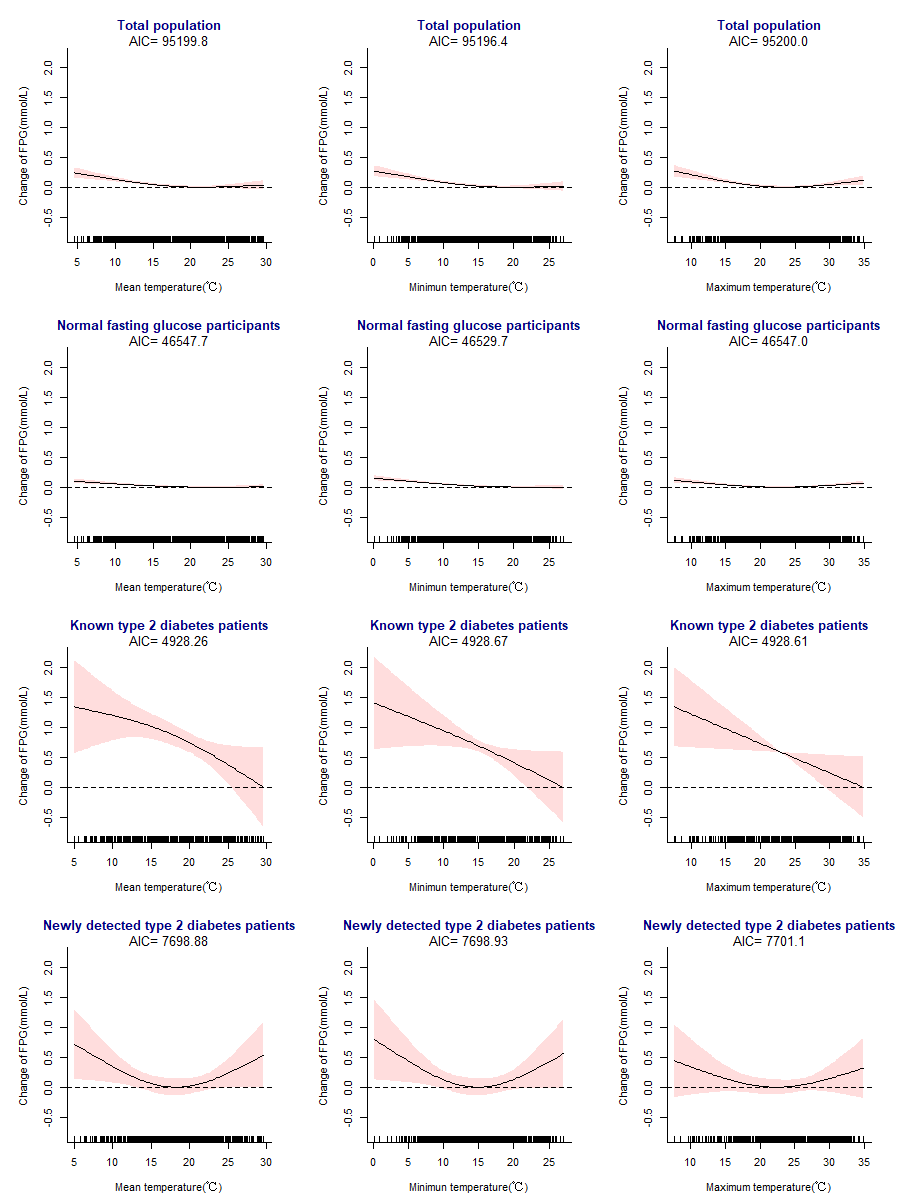


Figure S2. The non-linear relationships between daily mean, daily minimum, daily maximum temperature and FPG in total population and different T2DM subgroups.

Gaussian generalized additive mixed models were adjusted for age, sex, BMI, education, career, physical activity, sedentary leisure times, smoking status, drinking status, humidity and use of hypoglycemic medicine variables.


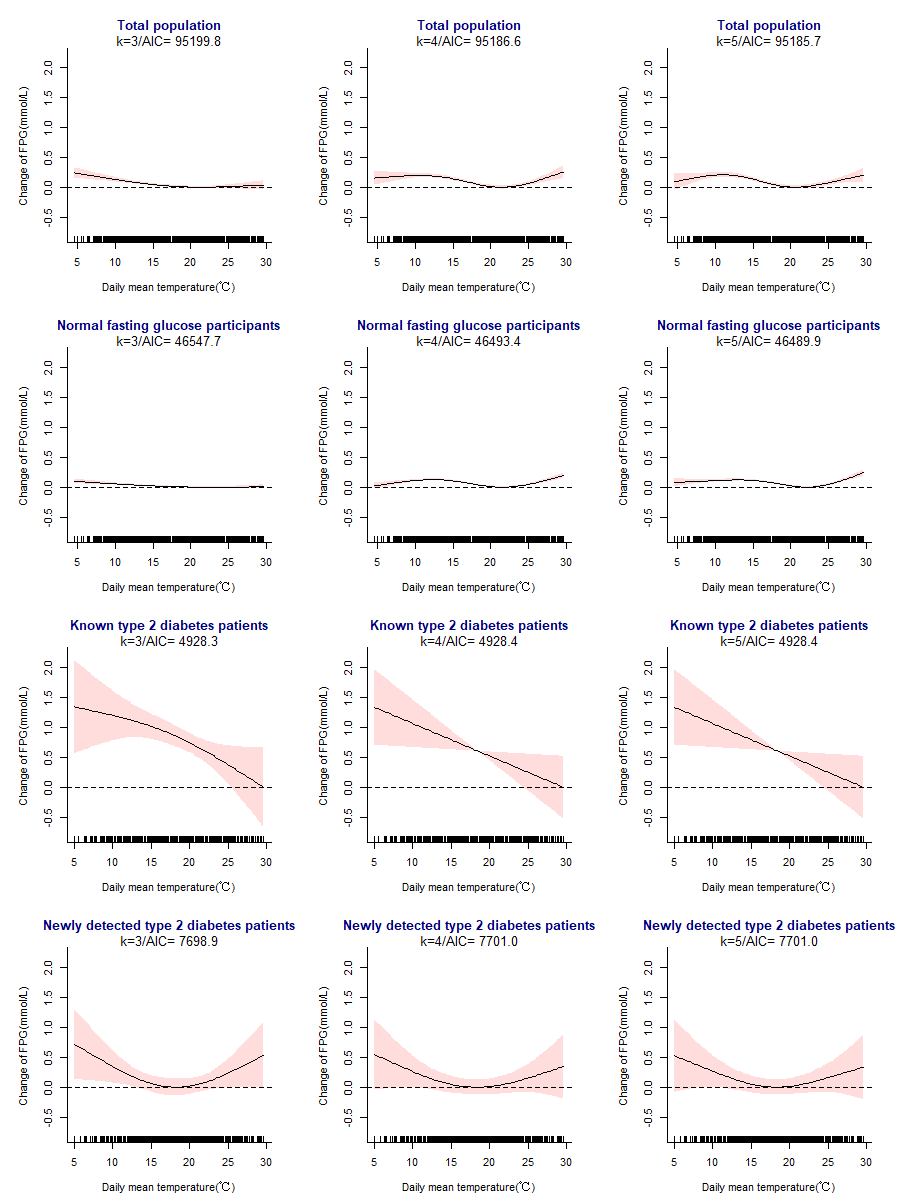
Figure S3. The non-linear relationships between daily mean temperature and FPG in total population and different T2DM subgroups at *k*=3,4,5.

Gaussian generalized additive mixed models were adjusted for age, sex, BMI, education, career, physical activity, sedentary leisure times, smoking status, drinking status, humidity and use of hypoglycemic medicine variables.
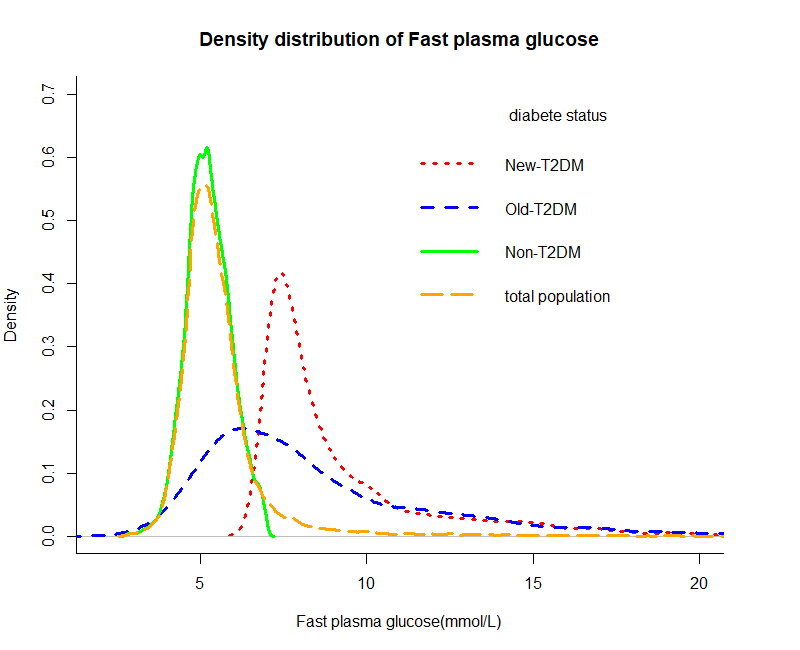


Figure S4. The density distribution of FPG in total population and different T2DM subgroups.

Table S2 Statistical description of fasting plasma glucose (FPG) and daily mean ambient temperature in total population and different T2DM subgroups.

|  | Mean | SD | P2.5 | P25 | P50 | P75 | P97.5 |
| --- | --- | --- | --- | --- | --- | --- | --- |
| **FPG (mmol/L)** |  |  |  |  |  |  |  |
| Total population | 5.59 | 1.56 | 4.01 | 4.83 | 5.30 | 5.86 | 9.58 |
| Normal fasting glucose participants† | 5.25 | 0.67 | 4.00 | 4.80 | 5.20 | 5.70 | 6.65 |
| Known T2DM patients‡ | 8.44 | 3.55 | 4.30 | 5.98 | 7.46 | 9.84 | 17.79 |
| Newly detected T2DM patients§ | 8.91 | 2.79 | 7.01 | 7.37 | 8.00 | 9.80 | 17.10 |
| **Daily mean Temperature (℃) ^#^** | |  |  |  |  |  |  |
| Total population | 18.20 | 5.44 | 7.64 | 14.62 | 18.17 | 21.94 | 28.12 |
| Normal fasting glucose† | 18.20 | 5.41 | 7.64 | 14.74 | 18.28 | 21.87 | 27.96 |
| Known T2DM patients‡ | 18.53 | 6.03 | 7.64 | 14.40 | 18.41 | 23.72 | 28.22 |
| Newly detected T2DM patients§ | 17.93 | 5.61 | 7.81 | 13.62 | 17.91 | 22.14 | 28.19 |

†: Normal fasting glucose participants, no medical history of type 2 diabetes mellitus with fasting plasma glucose<7.0mmol/L.

‡: Known T2DM patients, physician-diagnosed type 2 diabetes mellitus.

§: Newly detected T2DM, newly detected type 2 diabetes mellitus with fasting plasma glucose≥7.0mmol/L.

#: Daily mean Temperature was matched by the same date of health survey.

Table S3. Number (%), Mean and SD of fasting plasma glucose (FPG) and daily mean temperature by month.

| variable |  |  |  | Month |  |  |  |  |
| --- | --- | --- | --- | --- | --- | --- | --- | --- |
|  | January | February | March | April | May | October | November | December |
| Normal fasting glucose participants^†^, n (%) | 1559 (6.50) | 68 (0.30) | 605 (2.50) | 1276 (5.30) | 2963 (12.40) | 1783 (7.50) | 8636 (36.20) | 6987 (29.30) |
| FPG (mmol/L) | **5.55**±**0.65** | 5.50±0.51 | 5.29±0.61 | 5.30±0.61 | 5.34±0.67 | **5.05**±**0.62** | 5.15±0.66 | 5.30±0.67 |
| Daily mean Temperature^#^ (℃) | **10.98**±**3.27** | 13.34±2.14 | 18.80±2.69 | 22.76±3.00 | 26.69±1.40 | **22.29**±**2.16** | 18.74±2.78 | 13.82±3.68 |
| Known T2DM patients^‡^, n (%) | 91 (9.9) | 20 (2.20) | 30 (3.30) | 59 (6.40) | 205 (22.4) | 46 (5.00) | 243 (26.50) | 222 (24.20) |
| FPG (mmol/L) | 8.88±3.42 | 8.31±3.95 | **9.41**±**3.47** | 8.59±3.58 | 8.39±3.31 | **7.39**±**3.71** | 8.04±3.52 | 8.82±3.72 |
| Daily mean Temperature^#^ (℃) | 11.04±3.46 | 12.43±1.79 | **18.69**±**2.50** | 22.82±2.78 | 26.11±1.60 | **22.80**±**1.53** | 18.65±2.79 | 12.97±3.43 |
| Newly detected T2DM patients^§^, n (%) | 188 (12.10) | 15 (1.00) | 60 (3.90) | 85 (5.50) | 228 (14.60) | 85 (5.50) | 432 (27.70) | 464 (29.80) |
| FPG (mmol/L) | 9.09±2.69 | 9.09±2.04 | **10.04**±**3.01** | 9.06±2.63 | 9.37±3.01 | 9.15±3.02 | **8.99**±**2.70** | 9.13±2.79 |
| Daily mean Temperature^#^ (℃) | 11.62±3.12 | 13.72±1.64 | **18.72**±**2.63** | 22.97±3.08 | 26.39±1.56 | 22.35±2.31 | **18.70**±**2.93** | 14.05±3.64 |
| Total, n (%) | 1838 (7.00) | 103 (0.40) | 695 (2.60) | 1420 (5.40) | 3396 (12.9) | 1914 (7.30) | 9311 (35.30) | 7673 (29.30) |
| FPG (mmol/L) | 6.08±1.79 | **6.57**±**2.44** | 5.88±1.98 | 5.66±1.56 | 5.79±1.76 | **5.29**±**1.38** | 5.41±1.38 | 5.63±1.55 |
| Daily mean Temperature^#^ (℃) | 11.04±3.26 | **13.22**±**2.04** | 18.79±2.68 | 22.78±3.00 | 26.64±1.43 | **22.30**±**2.15** | 18.73±2.78 | 13.81±3.68 |

Data were expressed as mean±SD for continuous variables and as number (percentage) for categorical variables.

†: Normal fasting glucose participants, no medical history of type 2 diabetes mellitus with fasting plasma glucose<7.0mmol/L.

‡: Known T2DM patients, physician-diagnosed type 2 diabetes mellitus.

§: Newly detected T2DM patients, newly detected type 2 diabetes mellitus with fasting plasma glucose≥7.0mmol/L.

#: Daily mean Temperature was matched by the same date of health survey.


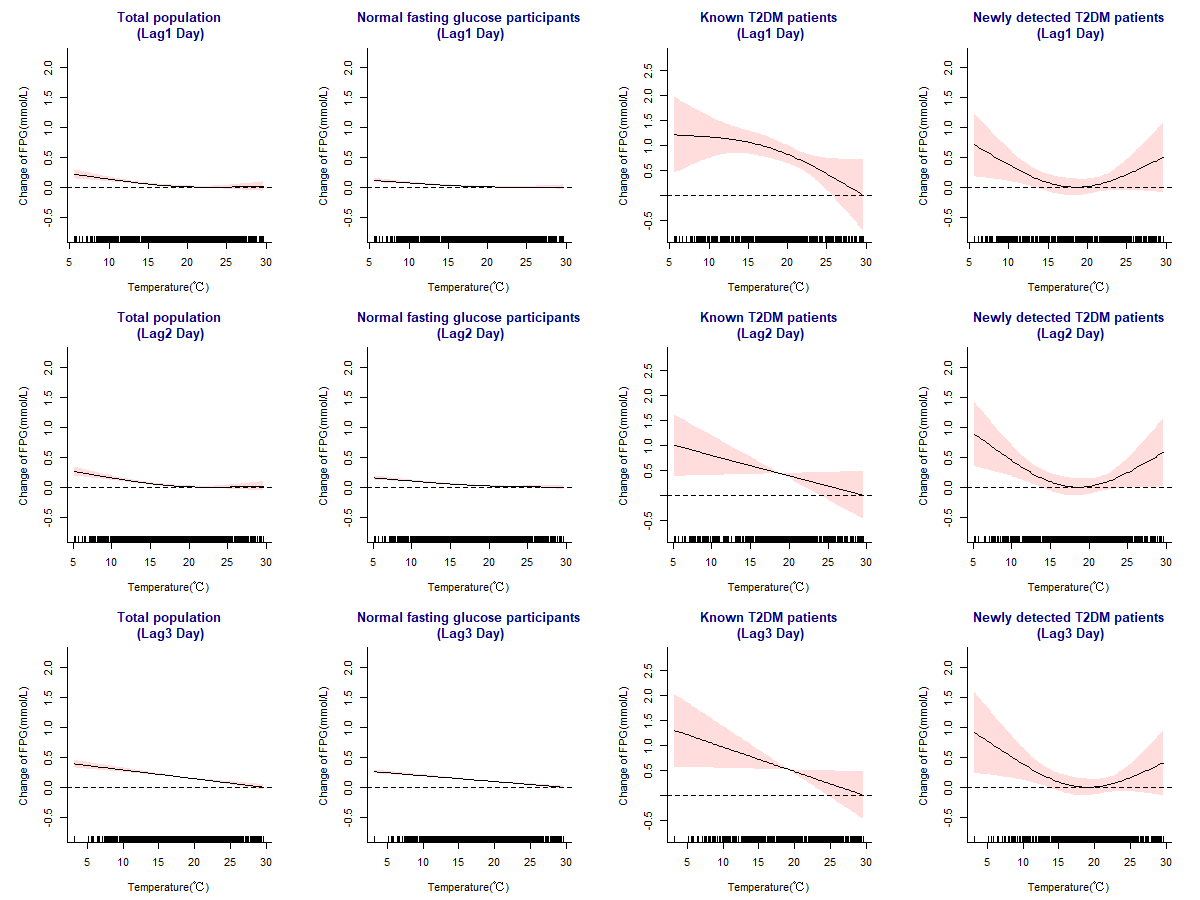


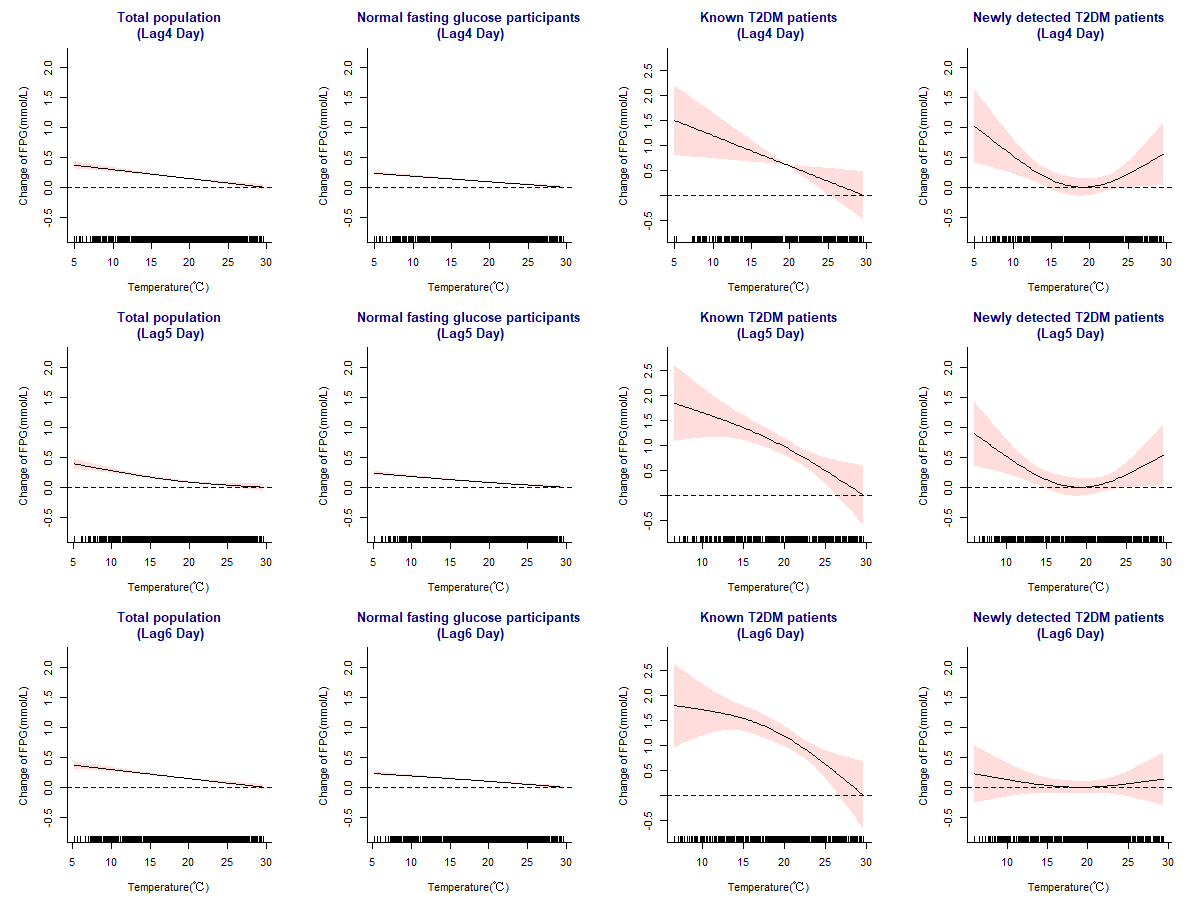
Figure S5. The relationships between lag1 to lag6 of ambient temperature and FPG in total population and different T2DM subgroups.

Gaussian generalized additive mixed models were adjusted for age, sex, BMI, education, career, physical activity, sedentary leisure times, smoking status, drinking status, humidity and use of hypoglycemic medicine variables.

Table S4 The interaction analyses about the association between temperature and FPG in total population based on effect modifier (T2DM status).

|  | Estimate and 95%CI | Interaction p-value |
| --- | --- | --- |
| T2DM status |  |  |
| Temperature*Normal fasting glucose | Ref | -- |
| Temperature*known T2DM | 0.098 (0.091, 0.105) | **<0.001** |
| Temperature*newly detected T2DM | 0.192 (0.189, 0.196) | **<0.001** |


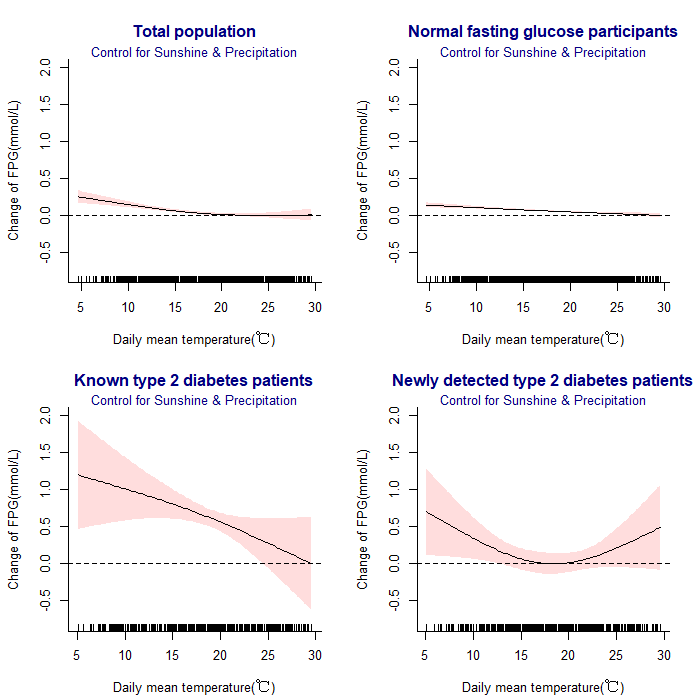
FigureS6. The relationships between ambient temperature and FPG in total population and different T2DM subgroups after additionally adjusting sunshine and precipitation.

Gaussian generalized additive mixed models were adjusted for age, sex, BMI, education, career, physical activity, sedentary leisure times, smoking status, drinking status, humidity, use of hypoglycemic medicine, sunshine and precipitation variables.


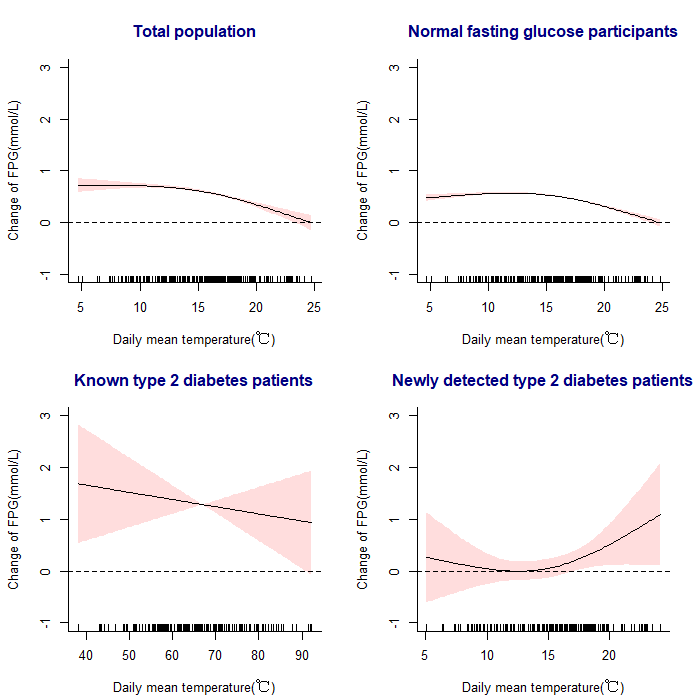
Figure S7. The relationships between ambient temperature and FPG in total population and different T2DM subgroups in survey of 2010 and 2015.

Gaussian generalized additive mixed models were adjusted for age, sex, BMI, education, career, physical activity, sedentary leisure times, smoking status, drinking status, humidity, sunshine, precipitation, use of hypoglycemic medicine, weekly consumption of grains, vegetables, fruit, meat and diabetes disease family variables.


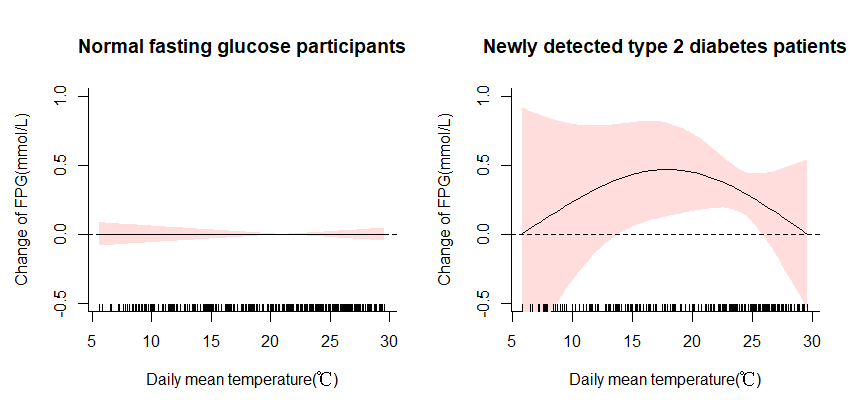
FigureS8. The relationships between ambient temperature and FPG in Normal fasting glucose participants and newly detected T2DM patients subgroups for the diagnostic standard of T2DM based on FPG and 2-hour plasma glucose in 2013 survey.

Gaussian generalized additive mixed models were adjusted for age, sex, BMI, education, career, physical activity, sedentary leisure times, smoking status, drinking status, humidity, use of hypoglycemic medicine, sunshine and precipitation.
